# Gonococcus infection probably acquired from bathing in a natural thermal pool: a case report

**DOI:** 10.1186/s13256-021-03043-6

**Published:** 2021-09-17

**Authors:** Felicity Goodyear-Smith, Robert Schabetsberger

**Affiliations:** 1 Department of General Practice & Primary Health Care, University of Auckland, PB 92019, Auckland, 1142 New Zealand; 2 Department of Biosciences, University of Salzburg, Salzburg, Austria

**Keywords:** Disease transmission, Infectious, *Neisseria gonorrhoeae*, Non-sexual transmission, Thermal pool, Case report

## Abstract

**Background:**

Authorities need to recognize that, while rare, gonorrhea can be transmitted nonsexually, and should not be presumed definitive evidence of abuse. We report the unusual case of a girl diagnosed with *Neisseria gonorrhoeae* after bathing in a heavily frequented hot pool at the edge of the crater lake Specchio di Venere (“Mirror of Venus”) on Pantelleria Island, Italy.

**Case presentation:**

Two days after bathing in the pool, this 11-year-old Austrian girl developed vulvovaginitis that partially settled with antifungal cream. Subsequent swabs cultured positive for *Neisseria gonorrhoeae.* Family members tested negative. The child adamantly denied any sexual contact, and no opportunities for sexual exposure could be identified. It was therefore concluded that she must have acquired the infection from pool water contaminated by gonococcus after a 2-day incubation period. The infection was successfully treated with ceftriaxone and azithromycin with no adverse effects.

**Conclusions:**

The pools are shallow, close to body temperature, isotonic, slightly acidic from CO_2_ bubbles, and contain organic particles, all potentially supporting survival of gonococcus. There are historical case reports in the literature of gonococcal epidemics in children’s hospitals being traced to common baths. It is imperative that all cases of gonococcal infection in children are fully investigated, including examining all other relevant family members, to determine whether sexual assault has occurred. This is not a diagnosis to be missed. However, both sexual and nonsexual transmission are possible. A presumption that a gonococcal infection is diagnostic of sexual abuse can be dire, with children wrongfully removed from their parents’ care, and their caregivers facing false charges of sexual crimes. Our case serves to illustrate that the very uncommon diagnosis of gonorrhea in a child may be the result of nonsexual transmission of the infection, and that contaminated hot pools are a very rare source of infection that should be considered.

## Background

*Neisseria gonorrhoeae* colonizes human mucosa, including conjunctiva, oropharynx, urethra, and anorectal tissues. Untreated, it may ascend into the female upper genital tract or into the male prostate and testis, and rarely, disseminated infection occurs in the form of septicemia or arthritis. While in adolescents and adult women the initial genital infection involves the cervix, prepubertal girls are susceptible to gonococcal vulvovaginitis. This is because the prepubertal vagina is slightly acidic to slightly alkaline (pH 6.5–7.5) with a thin atrophic mucosa. Postpubertal estrogenization produces a more acidic (pH 3.5–4.5) and keratinized vaginal epithelium, resistant to gonococcal colonization [1].

Gonococcus flourishes at 25–39 °C and is vulnerable to drying. Conditions for its optimal growth include humidity > 90%, isotonic environment, and 5–7% carbon dioxide at 36 °C [2]. *In vitro* experiments have cultured gonococcus from inoculated fabrics (towels and sheets) and other materials after at least several hours [3–6]. While gonococcal infection is predominantly sexually transmitted, numerous cases of accidental nonsexual transmission from autoinoculation, fomite transfer, or bathing in contaminated water are reported [3]. In individual cases, the possibility of sexual transmission cannot be excluded; however, the totality of the evidence is compelling support of occasional nonsexual transmission.

The aim of this paper is to report an unusual case of nonsexual transmission of gonorrhea probably acquired from bathing in a contaminated natural thermal hot pool.

## Case presentation

**Fig. 1:**
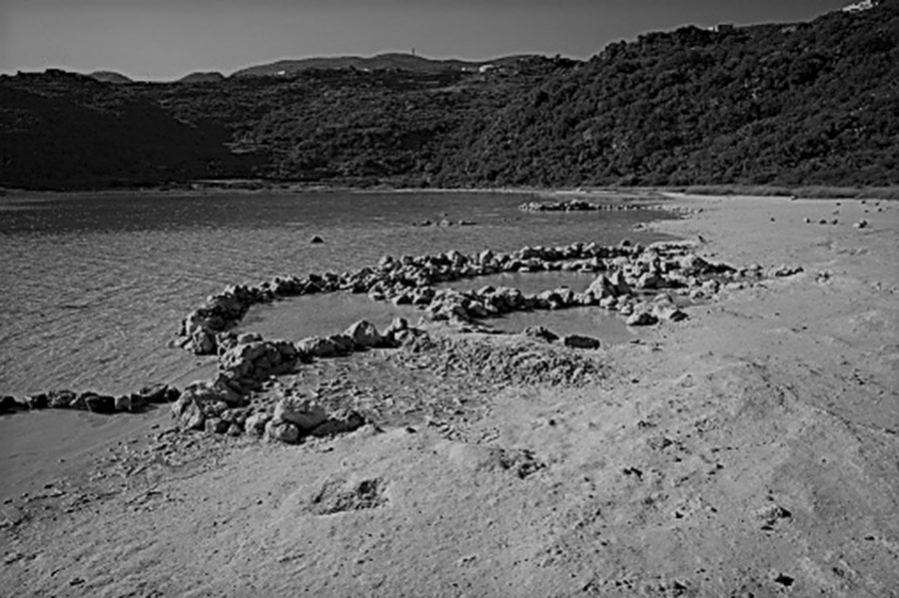
The shallow hot-spring pools created by stone walls on the edge of Lake Specchio Di Venere, Pantelleria Island, Italy (Permission to reproduce photograph provided by Onemag.it)

In August 2020, an 11-year-old Austrian girl visited the crater lake Specchio di Venere (“Mirror of Venus”) on Pantelleria Island, Italy with her parents and 7-year-old sister. After swimming in the lake, she spent an hour soaking in one of the hot pools at the lake’s edge (Fig. 1). She was lying in a pool about 20 cm deep and did not rinse off after leaving.

The pools are a major tourist attraction and are heavily used. There were five pools, with up to five people per pool, both adults and children, and frequent coming and going, with people waiting for a space to be vacated. The girl and her father were never alone in the pool. Her younger sister bathed in a different pool with her mother.

**Fig. 2:**
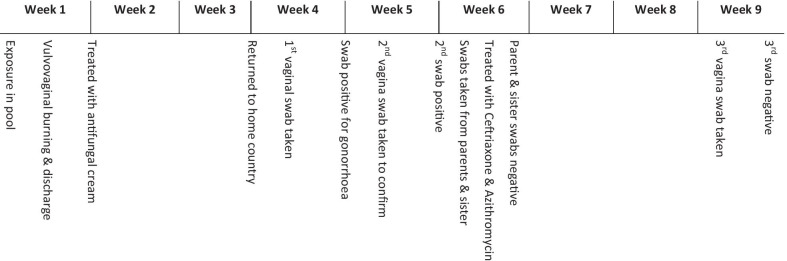
Case timeline

Two days after bathing in the pool, the girl developed vulvovaginal burning and discharge. The burning kept her from swimming in the sea for a couple of days, which caused disappointment whenever she tried to enter the water. Her symptoms partly settled but did not completely resolve with the use of antifungal cream purchased over the counter. The family returned to their home country Austria 2 weeks later, and she was then was seen by her pediatrician. She had no signs of sepsis, with no elevated temperature or pulse. Vaginal examination was normal, but a vaginal swab returned a positive aerobic culture for *Neisseria gonorrhoeae*. A second swab taken the following week confirmed the diagnosis of gonococcal vulvovaginitis. The parents and the younger sister were tested for gonorrhea with negative results (see Fig. 2 for the case timeline).

The possibility of sexual transmission was explored. The child adamantly denied any sexual contact. The family had been traveling together on holiday when the symptoms started, and there was no evidence or opportunity identified for sexual transmission. It was therefore concluded that she must have acquired the infection from pool water contaminated by gonococcus after a 2-day incubation period [7].

Once the diagnosis was established and the illness explained to her, her first reaction was fear that her new classmates might hear about this infection, as she had started high school immediately upon arrival in her hometown. The stress of adjusting to a new school coupled with the infection led to crying and fatigue, particularly just before treatment, as she was very afraid of the infusion.

However the pediatrician was gentle, both parents were present during treatment, and she coped well. She was treated with ceftriaxone infusion 50 mg/kg body weight followed by oral azithromycin 500 mg the next day. Her pediatrician also advised whey baths for 2 weeks to help restore her vaginal flora. Her vaginal symptoms settled within a couple of days. She experienced slight anorexia and loose stools for a couple of days post-treatment, but then made a full recovery. After being treated, she adapted well to her new school environment.

A repeat swab 4 weeks later was negative. Slight delays in getting swabs taken and treatment initiated occurred because the country was reentering lockdown for coronavirus disease 2019 (COVID-19), as well as the school year starting under pressure because of the imminent lockdown. She made an uneventful recovery and remains well.

## Discussion

We report the case of gonococcal vulvovaginitis in an 11-year-old prepubertal Austrian girl almost certainly contracted from bathing in a contaminated hot pool. It is imperative that all cases of gonococcal infection in children are fully investigated, including examining all other relevant family members, to determine whether sexual assault has occurred. This is not a diagnosis to be missed. However, both sexual and nonsexual transmission is possible. The consequences of the presumption that a gonococcal infection must be diagnostic of sexual abuse can also be dire, with children wrongfully removed from their parents’ care, and their caregivers facing false charges of sexual crimes.

The Specchio di Venere pools are shallow, murky, glass-green-colored from algae, and almost stagnant, although some water exchange occurs through the loosely stacked boulder walls. Hot water and gas bubbles (98% CO_2_) enter the pools through small holes in the sediment. Gradients in temperature develop within the pools from the source of the springs (> 40 °C) to where they mix with the lake water at the pools’ edges (~ 37°). Hence, the water is near body temperature, slightly acidic (pH 6.3–6.9), and closely isotonic (conductivity of 14–17 mS/cm at 25 °C), and contains mineral and organic particles as potential substrates for biofilms [8–10], potentially increasing the survival of gonococcus.

Prior to the advent of antibiotics, the source of gonorrhea epidemics in children’s hospitals was traced to common baths [11–13], as well as to towels, wash rags, diapers, bandages, bed linens, instruments, and children’s hands [13–24]. Epidemics of gonococcal conjunctivitis continue to occur in rural African and Australian aboriginal communities, thought to be mediated by flies, dirty fingers, and face cloths [25–27]. Case reports and cases series of probable household nonsexual transmission from sharing towels, clothes, and beds are also reported, from an index sexually transmitted case [28–49]. Unusual cases of nonsexual transmission include autoinoculation from a contaminated public toilet seat in an 8-year-old girl [50], and transmission from intercourse with an inflatable doll [51]. We report a case of vulvovaginal nonsexual transmission. This was presumably acquired from bathing in a shallow thermal pool frequented by many tourists, which is more likely than from using a public toilet. Hot pools have been recognized as sources of various serious infections [16, 17]. This rare event is likely due to a number of unique factors, including the timing of the child’s bathing in relation to that of an infected visitor, but those using these pools need to be alerted to the possibility of such exposure, including the risk of possible conjunctival infection, on occasion. It would be valuable to conduct experiments whereby water from the pools is inoculated with *Neisseria gonorrhoeae*, the temperature and CO_2_ aeration maintained, and then assays conducted to see if gonococcus can be cultured at different durations following inoculation, but this is beyond the scope of these authors.

There needs to be public understanding that people bathing in heavily frequented shallow thermal pools risk exposure to pathogens through inoculation by other bathers, including *Neisseria gonorrhoeae,* but also fecal contaminants such as *Escherichia coli* and *Pseudomonas* sp. We suggest provision of a shower and antibacterial soap near the hot springs. A sign should make visitors aware of strict hygiene before entering the pools.

The strength of this case report is that it adds to the literature by reporting a case of nonsexual transmission of *Neisseria gonorrhoeae* causing vulvovaginitis in a prepubertal child. The report draws on many other cases of nonsexual transmission reported in the literature, plus empirical published data demonstrating that the temperature, pH, and tonicity of the pools in question provide an environment in which gonococci might flourish. The case weakness is that, inevitably, it is impossible to prove that the pool was the definitive source of infection.

Gonococcal infections in prepubertal children occur very infrequently. Acquisition from sexual abuse must always be the first consideration, and should be investigated as a cause. However, authorities are reluctant to acknowledge that the mode of transmission may also be nonsexual, and that the infection is not definitive evidence of sexual assault. National guidelines may state that gonococcal infection in prepubertal children is always, or almost always, diagnostic of sexual abuse, without any discussion of possible nonsexual modes of transmission [7, 52, 53]. In her role as forensic physician, the lead author has encountered cases of nonsexually transmitted genital gonorrhea in prepubertal girls in Australia, New Zealand, the USA, Canada, and Denmark, where the child abuse authorities inform the courts that transmission can only be through some form of sexual activity with mucous membrane to mucous membrane contact. These cases include probable transmission from an infected mother using a towel to dry herself and then her 3-year-old daughter; an infected father sharing a towel with his 3-year-old daughter, and an 8-year-old girl using a wash cloth and towel immediately after her infected father had used them.

## Conclusion

Our case serves to illustrate that the very uncommon diagnosis of gonorrhea in a child may be the result of nonsexual transmission of the infection, as well as sexual, and that contaminated hot pools are a very rare source of infection that should be considered.

## Data Availability

Not applicable.
